# CD47 antibody blockade suppresses microglia-dependent phagocytosis and monocyte transition to macrophages, impairing recovery in EAE

**DOI:** 10.1172/jci.insight.148719

**Published:** 2021-11-08

**Authors:** Huan Wang, Gail Newton, Liguo Wu, Lih-Ling Lin, Amy S. Miracco, Sridaran Natesan, Francis W. Luscinskas

**Affiliations:** 1 Center for Excellence in Vascular Biology, Department of Pathology, Mass General Brigham and Harvard Medical School, Boston, Massachusetts, USA.; 2 Immunology & Inflammation Research Therapeutic Area, Sanofi US, Cambridge, Massachusetts, USA.

**Keywords:** Inflammation, Autoimmune diseases

## Abstract

Experimental autoimmune encephalomyelitis (EAE) is a well-characterized animal model of multiple sclerosis. During the early phase of EAE, infiltrating monocytes and monocyte-derived macrophages contribute to T cell recruitment, especially CD4^+^ T cells, into the CNS, resulting in neuronal demyelination; however, in later stages, they promote remyelination and recovery by removal of myelin debris by phagocytosis. Signal regulatory protein **α** and CD47 are abundantly expressed in the CNS, and deletion of either molecule is protective in myelin oligodendrocyte glycoprotein–induced EAE because of failed effector T cell expansion and trafficking. Here we report that treatment with the function blocking CD47 Ab Miap410 substantially reduced the infiltration of pathogenic immune cells but impaired recovery from paresis. The underlying mechanism was by blocking the emergence of CD11c^hi^MHCII^hi^ microglia at peak disease that expressed receptors for phagocytosis, scavenging, and lipid catabolism, which mediated clearance of myelin debris and the transition of monocytes to macrophages in the CNS. In the recovery phase of EAE, Miap410 Ab–treated mice had worsening paresis with sustained inflammation and limited remyelination as compared with control Ab–treated mice. In summary, Ab blockade of CD47 impaired resolution of CNS inflammation, thus worsening EAE.

## Introduction

Multiple sclerosis (MS) is a chronic inflammatory and demyelinating disease of the CNS with characteristic histopathological lesions that causes significant morbidity and mortality worldwide (1). Experimental autoimmune encephalomyelitis (EAE) is a preclinical animal model that approximates key pathological features of MS, including inflammation, demyelination, reactive microgliosis and astrocytic gliosis, and axonal loss (1–4). Rodent animal models have been integral to development of successful therapeutics for MS. In the CNS, extravasated immune cells and activation of resident CNS cells, including the microglia, oligodendroglia, and astrocytes, collectively have been shown to contribute to nerve demyelination and neuron injury, damage, and ultimately loss (reviewed in ref. 5).

CD47 is a broadly expressed type III transmembrane glycoprotein that interacts in the same plasma membrane in cis with leukocyte β_2_ integrins LFA-1 (CD11a/CD18) and Mac-1 (CD11b/CD18), and in trans with signal regulatory protein α (SIRPα, CD172a) and SIRPγ (CD172g) (6–10). SIRPα is expressed in myeloid leukocytes, glial cells, and neurons within the CNS and at low amounts in vascular endothelial cells (10–15). Binding of CD47 on circulating blood cells to SIRPα expressed on splenic myeloid cells transmits a negative “don’t eat me” inhibitory signal, preventing hematopoietic cell phagocytosis (16).

Previous studies have reported that CD47-knockout (*Cd47^–/–^*) mice were completely resistant to EAE induced by myelin oligodendrocyte glycoprotein aa 35–55 peptide (MOG) due to failed T effector cell expansion (17, 18). In contrast, the rat Ab against mouse CD47, Miap301, which partially blocked both the CD47 in cis interactions with LFA-1 and in trans CD47 interactions with SIRPα (19), did not reduce and instead worsened paresis through an unknown mechanism (18). In the same EAE model, another group recently reported that a murine CD47-Fc chimera molecule, which engages SIRPα and prevents in trans SIRPα/CD47 interactions, provides protection in EAE through a reduction in IL-1β–dependent recruitment of IL-17–producing effector T cells (20). Therefore, data on the effect of blocking the SIRPα/CD47 axis on EAE are incomplete. We reasoned that an Ab that specifically blocked the in trans CD47/SIRPα axis while not interfering with the in cis CD47/β_2_ integrin interactions might replicate the protection observed with a CD47-Fc chimeric molecule. We identified such a mouse Ab against mouse CD47, clone Miap410, which also cross-reacted with human CD47, that in our hands completely inhibited CD47/SIRPα interactions and not the in cis CD47/LFA-1 interaction required for T cell transendothelial migration in vivo and in vitro (19). Thus, we evaluated the therapeutic efficacy of Miap410 Ab in the EAE model.

In summary, treatment with Miap410 mAb substantially reduced the infiltration of pathogenic immune cells to the CNS but impaired recovery from paresis. The underlying mechanism was through blocking the emergence of CD11c^hi^MHCII^hi^ microglia expressing receptors for phagocytosis, scavenging, and lipid catabolism that mediated the clearance of myelin debris and the transition of monocytes to macrophages in the CNS. Thus, Miap410 treatment led to retention of proinflammatory lymphoid and myeloid leukocytes during the recovery phase. Of note is that Abs against CD47 that block the CD47/SIRPα axis are in multiple clinical trials as a cancer immunotherapeutic alone and in combination with other therapeutics and have shown efficacy in certain human cancers (21–25), and in reducing vascular inflammation of carotid arteries in a small retrospective analysis of patient trial data (26). Our results have wider implications beyond the EAE model and identify potential detrimental side effects on the CNS of treatments using CD47-blocking Ab that should be considered when treating patients with demyelinating diseases.

## Results

### MOG immunization induces paresis, immune cell infiltration, and demyelination of the spinal cords in C57BL/6 mice.

We developed multicolor flow cytometry panels to phenotype lymphocytic and myeloid immune cells isolated from spinal cords (SCs) of C57BL/6J mice immunized with MOG and sacrificed at the peak of disease and at the recovery phase and from nonimmunized (control), age-matched, female mice for comparison purposes based on the flow cytometric data of Caravagna and colleagues (27) (Supplemental Figure 1; supplemental material available online with this article; https://doi.org/10.1172/jci.insight.148719DS1).

**Figure 1:**
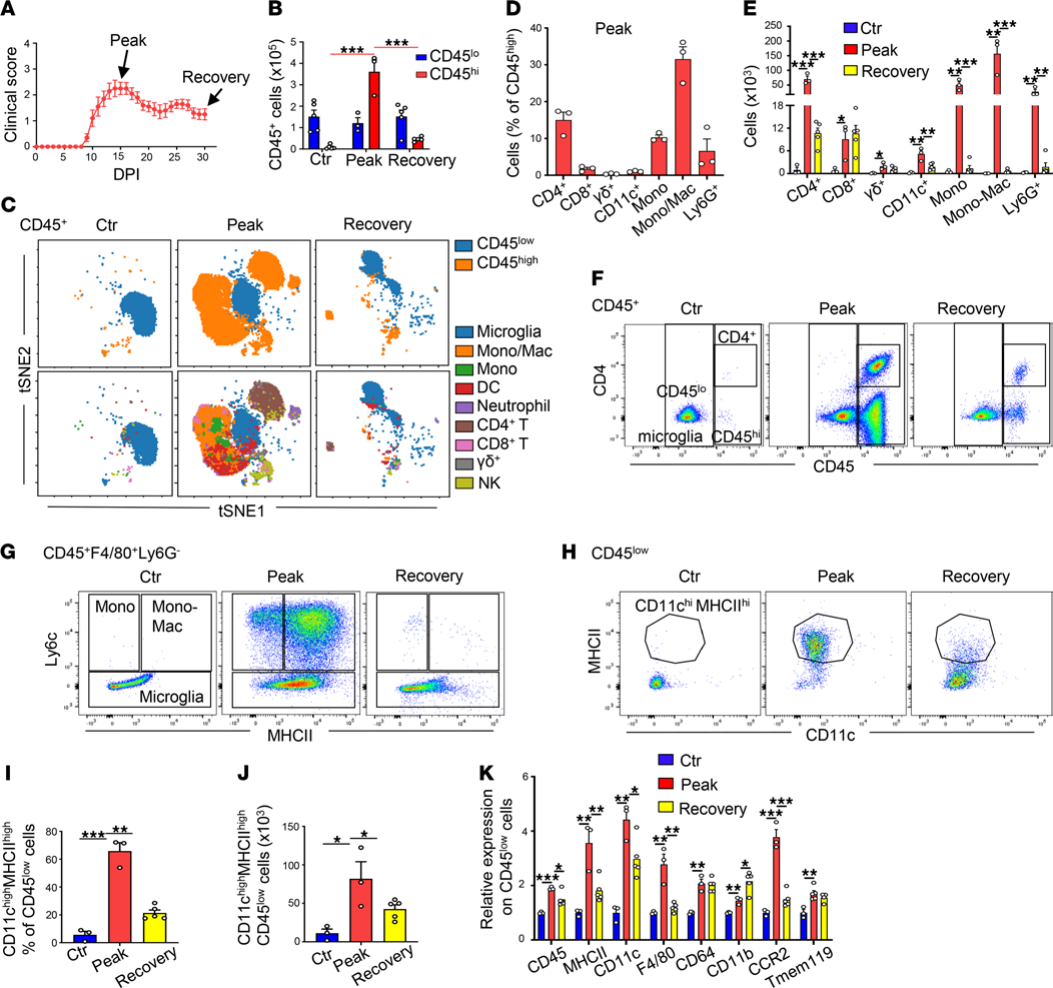
Phenotype of immune cells present in SCs of nonimmunized (control) and MOG-immunized mice by flow cytometry. (**A**) Clinical score (paresis). Data are shown as the mean ± SEM. *n* = 10 female C57Bl/6J mice. (**B**) Total number of CD45^+^CD45^lo^ (resident microglial cells) and CD45^hi^ (blood-derived) immune cells at peak disease (days postimmunization, DPI, 15), recovery phase (DPI 30) and in control nonimmunized mice. (**C**) t-Distributed stochastic neighbor embedding (t-SNE) representation of immune cells of EAE mice that cluster together. (**D**) Percentage of immune cell subtypes within CD45^hi^ cells at peak disease. (**E**) Total number of CD45^hi^ immune cell subtypes in SCs at peak and recovery phase and in nonimmunized control mice. (**F** and **G**) Representative plots of CD45^lo^ and CD45^hi^ cells, and CD4^+^ T cells within the CD45^+^ group of cells (**F**), and plots of monocytes (mono), monocyte-derived macrophages (Mono-Macs), and microglia within the CD45^+^F4/80^+^Ly6G^–^ group based on Ly6C and MHCII surface expression (**G**). (**H**) Representative plots of CD11c^hi^MHCII^hi^ cells within the CD45^lo^ group. (**I** and **J**) Percentage (**I**) and total number (**J**) of CD11c^hi^MHCII^hi^ within the CD45^lo^ group. (**K**) Relative surface expression of markers on CD45^lo^ resident microglia normalized to control mice. All data were from SCs. Data are shown as mean ± SEM. *n* = 3–5 mice per group. *P < 0.05, **P < 0.01, ***P < 0.001. A 1-way ANOVA with Tukey’s post hoc test was used to determine the statistical significance.

Mice immunized with MOG developed EAE disease as illustrated in Figure 1A. SCs harvested from mice at peak paresis scores contained a large number of immune cells that separated into 2 distinct populations based on CD45 leukocyte antigen staining: a large population of blood-derived CD45^hi^ leukocytes and a smaller population of resident microglia CD45^lo^ immune cells (Figure 1, B and C). The CD45^hi^ population dropped off by 88% (*P* < 0.001) at recovery DPI 30, while the total number of CD45^lo^ microglia was unchanged (Figure 1B). Studies by multiple groups have shown that CD45^hi^ immune cells are recruited from the peripheral circulation into the SCs while the CD45^lo^ cells are identified as resident Tmem119^+^ microglia (27–30). The identities of CD45^lo^ and CD45^hi^ immune cells, as well as subtypes in CD45^hi^ cells, were represented by use of t-SNE plots derived from the flow cytometric data (Figure 1C). Moreover, the heatmap of t-SNE showed that SIRPα was highly expressed on blood-derived myeloid cells and resident microglia as compared with T cells in the SCs at peak disease (Supplemental Figure 2).

Focusing on the CD45^hi^ population, 7 major cell clusters were identified and represented about 70% of the total cell number at disease peak. These cell types were CD4^+^ T cells (15.1%), CD8^+^ T cells (1.8%), γ/δ T cells (0.4%), F4/80^+^Ly6C^hi^MHCII^lo^ blood-derived monocytes (10.3%), F4/80^+^Ly6C^hi^MHCII^hi^ monocyte-derived macrophages (Mono-Macs) (31.5%), CD11c^hi^MHCII^hi^ dendritic cells (DCs) (1.1%), and Ly6G^+^ neutrophils (6.6%), as defined by multicolor flow cytometry (Figure 1D; see Supplemental Figure 1 for the gating scheme for flow cytometry analysis). The numbers of each cell type at peak disease dropped off significantly in the recovery phase of EAE (Figure 1E). CD4^+^ T cells that play a central role in EAE development were recruited in significant numbers from circulating blood (CD45^hi^) to the SCs and dropped off dramatically in the recovery phase (Figure 1, C, E, and F). Another gating population of CD45^+^F4/80^+^Ly6G^–^ cells in SCs at peak disease consisted of CD45^hi^ monocytes, Mono-Macs from circulating blood, and the CD45^lo^ microglia (Supplemental Figure 3). Robust numbers of Ly6C^hi^ monocytes and Mono-Macs invaded into the SCs at the peak of disease and dramatically declined at the recovery phase (Figure 1, E and G).

Analysis of the CD45^lo^ microglia at disease peak revealed the emergence of a new population of CD11c^hi^MHCII^hi^ microglia (Figure 1, H–J). This microglia population at the peak of disease showed dramatic increases in the surface expression of several myeloid markers, suggesting an activated phenotype (MHCII, F4/80, CD64, and CD11b and CD11c integrins) compared with a nonimmunized control (Figure 1K). Importantly, these CD11c^hi^MHCII^hi^ microglia depicted in Figure 1H and phenotyped in Figure 1J recently have been shown to provide reparative functions through autophagy-mediated phagocytosis and clearance of myelin debris and the removal of injured oligodendrocytes in the CNS during the recovery of EAE or cuprizone-induced demyelination (31–35). At recovery on DPI 30, the CD11c^hi^MHCII^hi^ microglia dropped by 50% (Figure 1, I and J). The surface expression of CD45, MHCII, CD11c, CCR2, and F4/80 also was decreased (Figure 1K).

**Figure 2:**
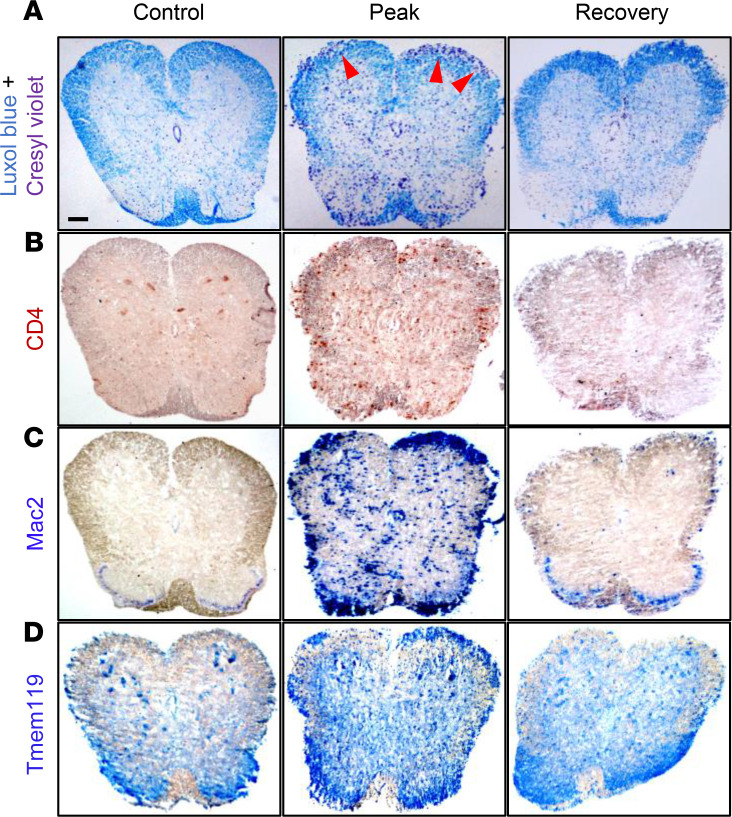
Histological staining of immune cells in SCs of EAE mice. (**A**) Representative images of Luxol fast blue staining of SCs in control nonimmunized mice (Control), EAE mice at peak disease (DPI 15), and EAE mice at recovery phase (DPI 30) from 3 mice per group that had clinical scores similar to the group’s paresis score. Red arrowheads identify regions of inflammation and demyelination of SC white matter. (**B**–**D**) Representative images of CD4^+^ T cells (**B**), Mac2^+^ macrophages (**C**), and Tmem119^+^ microglia (**D**) staining in SCs of control and EAE mice. Scale bar equals 200 μm.

We complemented the flow cytometric analyses with immunohistology assessment of SCs from MOG-immunized and control mice. The SCs from immunized mice at peak disease showed clear histological evidence of demyelination in regions of the white matter by Luxol blue/cresyl violet staining that colocalized with CD4^+^ T cells and a massive infiltrate of monocytes/macrophages positive for Mac2 antigen (galectin-3) as compared with SCs from control mice (Figure 2, A–C; red arrowheads). Consistent with the flow cytometry analysis, there was visible histological upregulation of Tmem119 expression on microglia in the peripheral white matter at peak disease compared with control (Figure 2D). At recovery, the inflammation was reduced, and the restoration of myelin in the white matter had increased. Thus, the histopathological features of MOG-induced inflammation at both the disease peak and recovery phases are consistent and corroborate the flow cytometry data in Figure 1.

### CD47 Ab Miap410 reduces immune cell infiltration into SCs and delays disease onset but causes sustained paresis.

**Figure 3:**
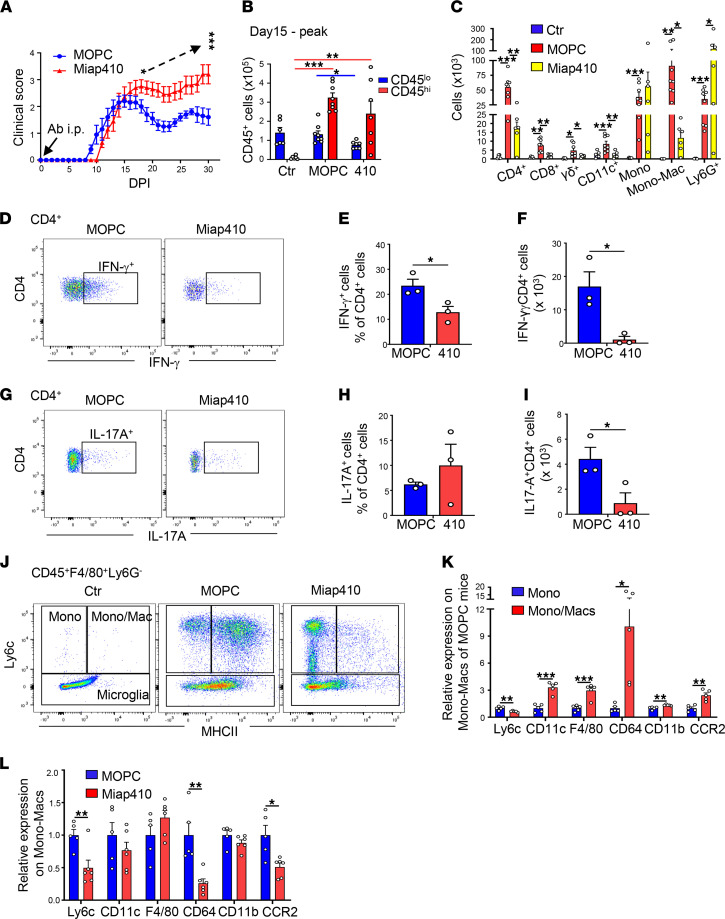
CD47 Ab Miap410 reduces immune cell infiltration into SCs and delayed EAE onset but worsens paresis. (**A**) Clinical scores. Ab treatments started on DPI 0. Data are shown as the means ± SEM. *n* = 10 mice per group. (**B**) Total number of different CD45^hi^ immune cells at peak disease (DPI 15) in Miap410 Ab– and MOPC-21 Ab–treated mice compared with nonimmunized (Ctr) mice. *n* = 6–8 mice per group. (**C**) Total number of different immune cell types. *n* = 6–8 mice per group. (**D**–**I**) Representative plots, and the percentage and total number of the CD4^+^ T cells producing IFN-γ (**D**–**F**) or producing IL-17A (**G**–**I**), at peak disease. *n* = 3 mice per group. (**J**) Representative plots of microglia, monocytes, and Mono-Macs within the CD45^+^F4/80^+^Ly6G^–^ group of cells based on Ly6C and MHCII surface expression at peak disease compared with nonimmunized (Ctr) mice. Representative plots of CD45^lo^ and CD45^hi^ cells. (**K**) Relative protein expression on monocytes and Mono-Macs from MOPC-21 Ab–treated mice at peak disease. (**L**) Relative protein expressions on Mono-Macs from MOPC-21– and Miap410-treated mice at peak disease. *n* = 4–6 mice per group. All data were from SCs. Data are shown as mean ± SEM. *P < 0.05, **P < 0.01, ***P < 0.001. One-way ANOVA with Tukey’s post hoc test was used (**B** and **C**). Paired Student’s *t* test (**A**) and unpaired Student’s *t* test (**E**, **F**, **H**, **I**, **K**, and **L**).

To test the hypothesis that blockade of the in trans CD47/SIRPα axis without affecting CD47-regulated LFA-1 integrin function is therapeutic in EAE development and recovery, cohorts of mice were immunized with MOG and treated with CD47 Ab Miap410 or with the isotype control Ab MOPC-21 every 48 hours beginning the same day as MOG immunization (DPI 0). Administration of Miap410 Ab delayed disease onset by 2 days; however, continued Ab treatment increased and sustained the disease scores compared with MOPC-21 treatment (Figure 3A). To gain insight into the cause of worsening disease, SCs from Ab-treated mice at peak of disease and at the recovery phase were subjected to flow cytometric and immunohistology analyses. We hypothesized that worsening disease in Miap410-treated mice was due to either an increased influx of proinflammatory encephalitogenic T cells, monocytes, and macrophages, or a reduction of reparative (phagocytic) Mono-Macs and CD11c^hi^MHCII^hi^ microglia that previously have been shown to resolve inflammation in the EAE model (28, 33).

The SCs isolated at peak disease from Miap410 Ab–treated mice showed a strikingly different content of immune cells and phenotype as compared with mice treated with MOPC-21 Ab. First, Miap410 significantly reduced the influx of most blood-derived CD45^hi^ leukocytes. The numbers of T cells (CD4^+^, CD8^+^, and γ/δ T cells) and CD11c^+^ DCs were reduced by 88% (*P* < 0.01) in Miap410-treated mice, even though there was no statistical difference in the total number of CD45^hi^ cells in SCs from either treatment group (Figure 3, B and C). On the other hand, Ly6G^+^ neutrophils were significantly elevated by Miap410 Ab, but quite variable, suggesting the presence of acute injury or necrosis in some of the Miap410-treated mice (Figure 3C). Previous studies have implicated both IL-17–producing T helper (Th17) cells and IFN-γ–producing T helper cell type 1 (Th1) cells in the pathogenesis of MOG-induced EAE (27, 36). Miap410 Ab significantly reduced the recruitment of CD4^+^ T cells (Figure 3C). Analysis of CD4^+^ T cell cytokine production revealed Miap410 significantly reduced the numbers of Th1 and Th17 T cells in SCs compared with MOPC-21 (Figure 3, D–I). Other T cell subsets were present albeit at much lower numbers (<3000 cells/SC), which included IL-17^+^ γ/δ T cells, CD8^+^IFN-γ^+^ T cells, CD4^+^FoxP3^+^ T regulatory cells, and NK1.1^+^ cells. The differences in cell numbers were not statistically significant from MOPC-21 Ab–treated mice (Supplemental Figure 4, A–D). Second, the number of monocytes in the SCs was slightly but significantly increased in Miap410 mice; however, dramatically fewer Mono-Macs (94% reduction, *P* < 0.05) were detected in Miap410- compared with MOPC-21–treated mice (Figure 3, C and J). Third, there were significant differences in the expression of surface molecules between monocytes and Mono-Macs in the MOPC-21–treated groups (Figure 3K). The higher expression of CCR2 on Mono-Macs in MOPC-21 mice may have aided localization to sites of injury to facilitate clearance of extracellular myelin and debris as previously reported (37). Furthermore, Miap410 treatment markedly limited the increase in surface expression of Ly6C, CD64, and CCR2 on Mono-Macs (Figure 3L). Collectively, the major consequences of Miap410 Ab were the dramatic block in the recruitment of pathogenic immune cells, especially cytokine production of Th1 and Th17 effector T cells, and the block in monocyte transition to macrophages. These data indicate that worsening disease in Miap410-treated mice was not due to a massively increased influx of proinflammatory encephalitogenic T cells, monocytes, or Mono-Macs. We hypothesized, therefore, that worsening disease by Miap410 Ab was due to the impaired emergence of reparative macrophages and/or CD11c^hi^MHCII^hi^ microglia, given that both cell types were required to resolve CNS inflammation based on prior studies (28, 33, 38, 39).

### CD47 Ab Miap410 reduces the emergence of CD11c^hi^MHCII^hi^microglia in SCs, impairing the recovery of EAE mice from paresis.

**Figure 4:**
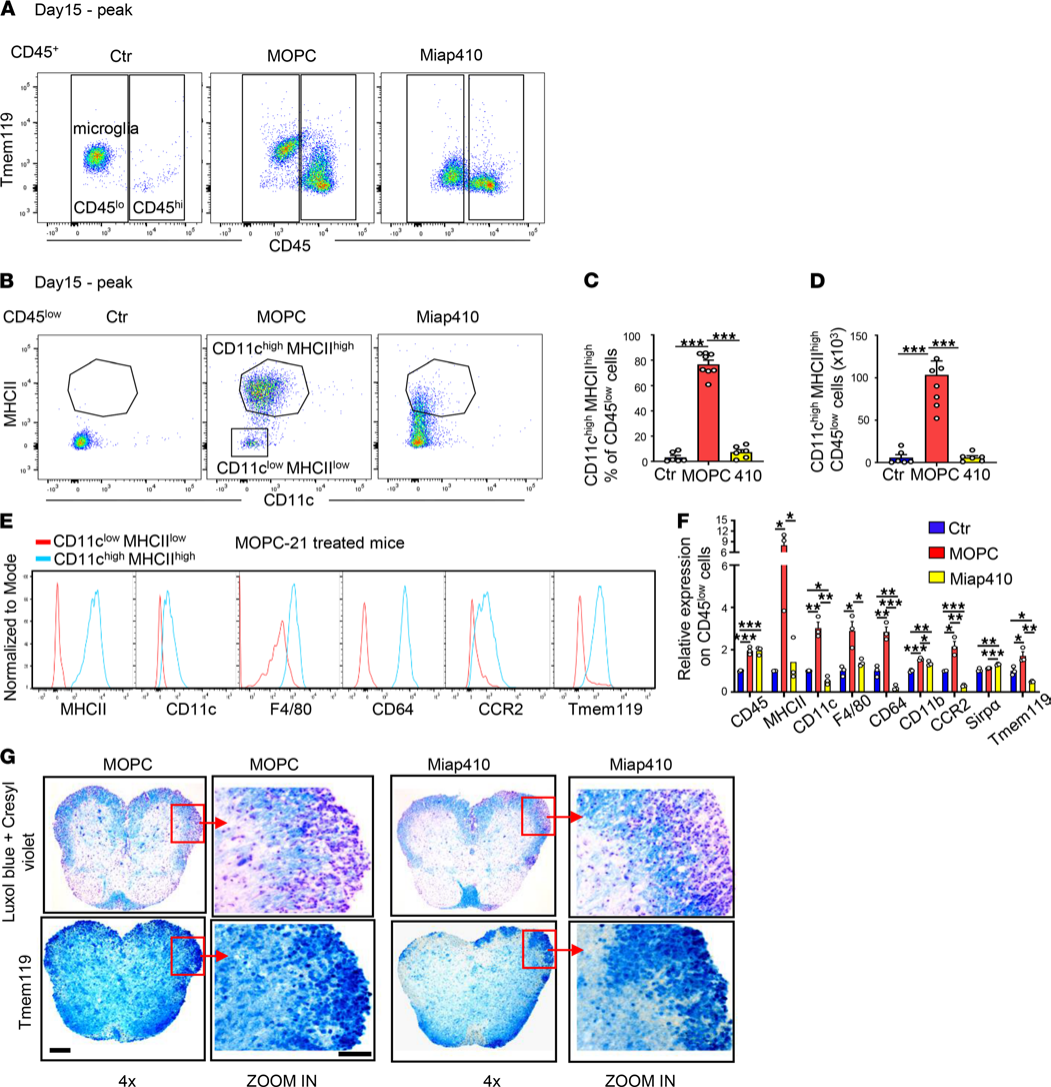
CD47 Ab dramatically reduces CD45^lo^CD11c^hi^MHCII^hi^ microglial cells in SCs at peak disease. (**A**) Representative plots of CD45^lo^ cells in SCs and gating on CD11c^hi^MHCII^hi^ microglia at peak disease in Ab-treated and nonimmunized control (Ctr) mice. (**B**–**D**) Representative plot of CD11c^hi^MHCII^hi^ (**B**) and their percentage (**C**) and total number (**D**) within the CD45^lo^ cell population. *n* = 6–7 mice per group. (**E** and **F**) Representative plots (**E**) and normalized surface expression of antigens (**F**) on CD45^lo^ cells from SCs of Ctr, MOPC-21, and Miap410 Ab–treated mice. *n* = 3 mice per group. (**G**) Representative images of Luxol fast blue and Tmem119 antigen staining in SCs of EAE mice. Scale bars represent 200 μm in SCs and 100 μm for zoomed-in images. Images are representative of *n* = 3 mice per group. All data from SCs. Data are shown as mean ± SEM values. *P < 0.05, **P < 0.01, ***P < 0.001. One-way ANOVA with Tukey’s post hoc test was used for the statistical significance.

Our study demonstrates that Miap410 treatment compared with MOPC-21 Ab resulted in a dramatic drop in the total number of Tmem119^+^CD45^lo^ microglia and a striking 95% reduction in CD11c^hi^MHCII^hi^ microglia in the SCs at peak disease (Figure 4, A–D, and Figure 3B). Moreover, our observations from MOPC-21–treated mice at peak disease showed significantly higher surface expression of F4/80, CD64, CCR2, and Tmem119 molecules in the CD11c^hi^MHCII^hi^ microglia than the CD11c^lo^MHCII^lo^, suggesting CD11c^hi^MHCII^hi^ microglia were activated and accelerated the reparative process within inflamed SCs (Figure 4E). This scenario is consistent with previous reports that CD11c^hi^ microglia are considered “primed,” or in a preactivation state, and appear during remodeling of myelin during a late developmental stage or accumulate at sites of damaged myelin debris during aging (38, 40). In contrast to control MOPC-21 Ab, Miap410 treatment induced a striking failure (95% reduction) in the expansion of CD11c^hi^MHCII^hi^ microglia and failed upregulation of these important molecules (Figure 4, B and F). Immunohistological images of Tmem119 Ab staining, which identifies microglia (41), revealed that their numbers were dramatically reduced in Miap410-treated mice compared with MOPC-21 (Figure 4G). This result corroborates the flow cytometry data in Figure 4A and Figure 3B. Despite these defects due to Miap410 Ab treatment, the Tmem119^+^ microglia still localized to demyelinated regions in both treatment groups, as demonstrated in Figure 4G.

**Figure 5:**
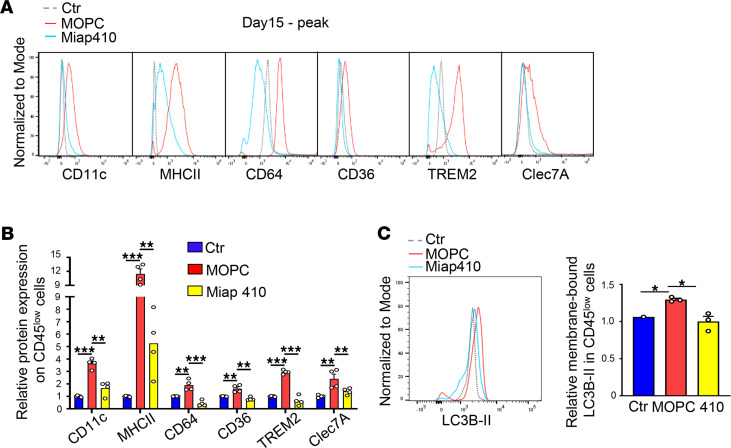
CD47 Ab Miap410 reduces phagocytosis-related proteins on microglia in SCs of EAE mice. (**A**) Representative plots of phagocytosis-, scavenger-, and lipid catabolism–related proteins on CD45^lo^ cells in Ab-treated mice and nonimmunized (Ctr) mice. (**B**) Relative surface expression of phagocytosis-related proteins on CD45^lo^ cells from Ab-treated or nonimmunized (Ctr) mice. (**C**) Representative plots and relative membrane-bound LC3B-II expression in CD45^lo^ cells from nonimmunized (Ctr) and Ab-treated mice. All data from SCs. Data are shown as means ± SEM. *n* = 3–5 mice per group. *P < 0.05, **P < 0.01, ***P < 0.001. One-way ANOVA with Tukey’s post hoc test was used for the statistical significance.

Furthermore, we observed that microglia from SCs of Miap410 Ab–treated mice showed significantly reduced expression of activation markers, CD11c and MHCII, and phagocytic function molecules CD64, CD36, TREM2, and Clec7A, as compared with MOPC-21 mice (Figure 5, A and B). In addition, the expression of membrane-bound LC3B-II also was significantly lower in microglia from Miap410-treated mice, suggesting diminished autophagy activity might have occurred compared with MOPC-21 Ab (Figure 5C). These results suggest that Ab blockade of CD47 in EAE mice worsened disease, in part, because of the inhibition of both microglia activation and phagocytosis- and/or autophagy-mediated removal of myelin debris in the SCs.

### CD47 Ab Miap410 sustains inflammation and severe loss of myelin in the white matter of SCs at the recovery phase.

**Figure 6:**
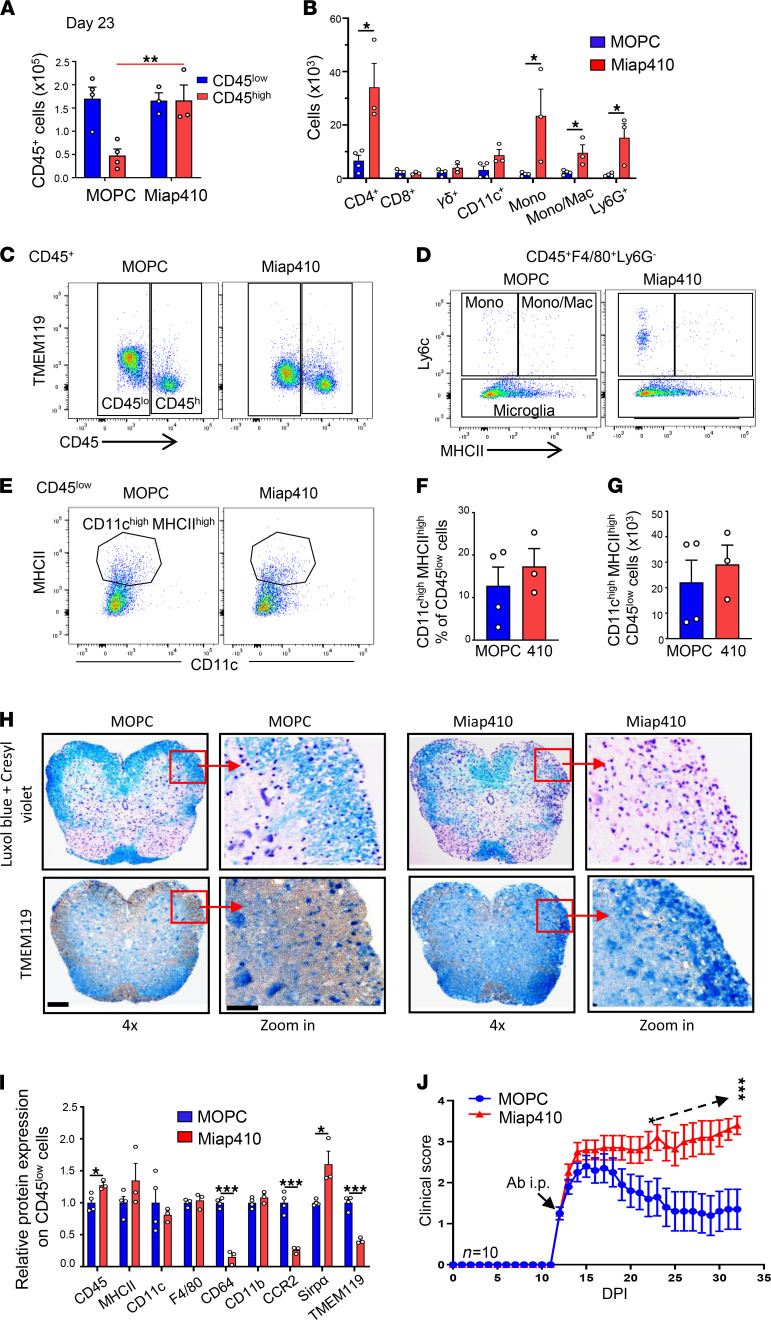
CD47 Ab Miap410 prevented recovery of mice from EAE compared with isotype control Ab MOPC-21. (**A**) Total number of CD45^+^, CD45^lo^ (microglia), and CD45^hi^ (blood-derived) immune cells at recovery phase (DPI 23) of EAE. (**B**) Total number of immune cell subtypes at recovery phase. (**C**–**E**) Representative plots of CD45^lo^ and CD45^hi^ within CD45^+^ cells (**C**), monocytes/macrophages/microglia within CD45^+^F4/80^+^Ly6G^–^ cells (**D**), and CD11c^hi^MHCII^hi^ cells within CD45^lo^ cells (**E**). (**F** and **G**) Percentage (**F**) and total number (**G**) of CD11c^hi^MHCII^hi^ within CD45^lo^ cells in SCs at recovery phase. (**H**) Representative images of Luxol fast blue and Tmem119 staining of SCs from 3 mice of similar average clinical score. Scale bars represent 200 μm in SCs and 100 μm for zoomed images. (**I**) Relative surface marker expression on CD45^lo^ cells isolated from SCs of Ab-treated mice. *n* = 3–4 per group. (**J**) Clinical score of an R-EAE mouse model given anti-CD47 Ab Miap410 or control Ab MOPC-21 every 48 hours starting at an average score of 1 (12 DPI). Data are shown as means ± SEM. *n* = 10 female SJL mice. *P < 0.05, **P < 0.01, ***P < 0.001. Unpaired Student’s *t* test was used (**A**, **B**, **F**, **G**, and **I**) and paired Student’s *t* test (**J**).

Next, we investigated the phenotype of immune cells and histological features of the SCs isolated from mice during the recovery phase of EAE (DPI 23). Not surprisingly, the SCs of mice treated with Miap410, compared with MOPC-21 Ab–treated mice, contained significantly greater numbers of CD45^hi^ leukocytes that consisted of primarily CD4^+^ T cells and variable numbers of monocytes, Mono-Macs, and neutrophils (Figure 6, A–D). The presence of increased CD4^+^ T cells and DCs suggests a continued MOG-driven CD4^+^ T cell activation and proliferation and/or entry of naive T cells and their activation by local antigen-presenting cells to initiate epitope spreading that has been considered a major cause of disease progression in murine encephalomyelitis models (42). On the other hand, the number of CD11c^hi^MHCII^hi^ microglia was similar in both Miap410- and MOPC-21–treated mice at the recovery phase (Figure 6, E–G); however, in the MOPC-21 Ab group, the actual number of microglia was markedly reduced compared with the number at the peak of disease (Figure 6G vs. Figure 4D). The microglia in Miap410 Ab– compared with MOPC-21 Ab–treated mice were quantitatively a less activated phagocytic and reparative phenotype (Figure 6I). Histopathology images of Luxol blue staining revealed a dramatically lower number of myelinated neurons in the white matter of SCs from Miap410 Ab mice compared with MOPC-21 mice, and these lesions contained significant numbers of Tmem119^+^ microglia (Figure 6H). These data suggest that Miap410 Ab treatment caused sustained CD4^+^ effector T cell–mediated inflammation and impaired remyelination in the white matter of SCs in EAE.

In the clinic, individuals with undiagnosed MS do not receive treatment until the symptoms occur and are correctly diagnosed. Therefore, the impact of Miap410 Ab treatment on the progression of existing EAE is desired. To further determine the effect of Miap410 Ab on existing EAE, we initiated administration of Miap410 Ab at clinical score 1 in the relapsing EAE (R-EAE) SJL mouse model that mimics the EAE disease characterized by a relapsing-remitting course of paralysis induced by proteolipid protein peptide aa 139–151, which allows assessment of the efficacy of various immunoregulatory strategies in a progressive autoimmune disease setting. This study revealed that Miap410-initiated treatment at the clinical score 1 caused severe and sustained paresis in the R-EAE mouse model. These data mirrored the effect on disease scores observed in MOG-induced EAE (Figure 6J compared with Figure 3A).

### Microglia in nonimmunized mice were differently affected by CD47 Miap410 Ab treatment compared with genetic deficiency in CD47.

**Figure 7:**
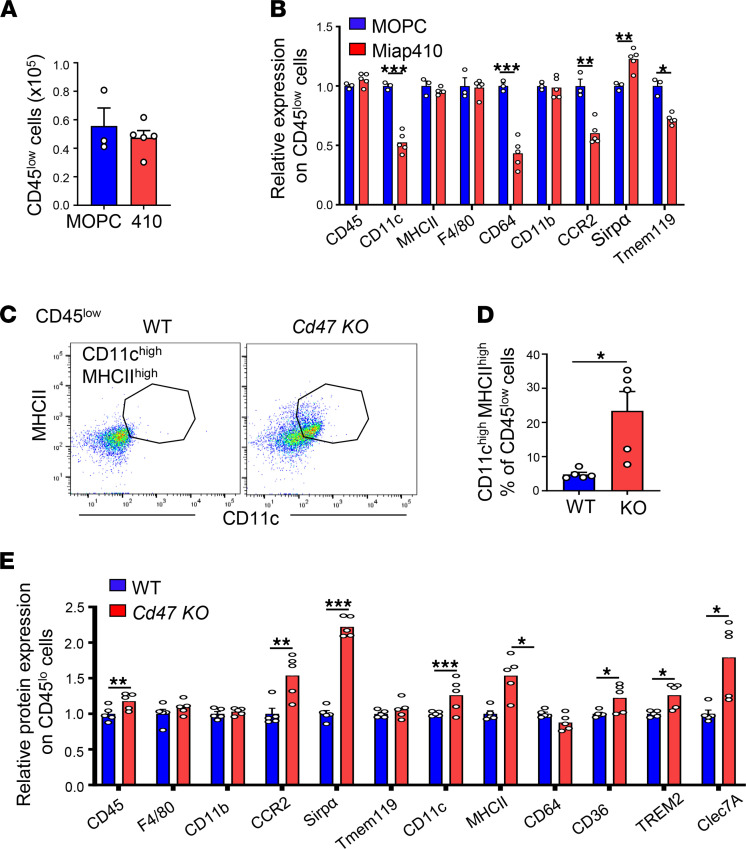
Microglia phenotype is differentially affected by CD47 Ab Miap410 treatment and genetic deletion of CD47. (**A**) Total number of CD45^lo^ microglia cells in SCs after 7 injections (i.p.) of Miap410 or MOPC-21 Ab. (**B**) Relative surface expression of key molecules on CD45^lo^ cells from SCs of Ab-treated mice. (**C**) Representative plots of CD11c^hi^MHCII^hi^ cells within CD45^lo^ cell population in *Cd47^–/–^* and control mice (WT). (**D**) Percentage of CD11c^hi^MHCII^hi^ cells within CD45^lo^ cell population in *Cd47^–/–^* and WT mice. (**E**) Relative surface expression on CD45^lo^ cells from SCs of *Cd47^–/–^* and WT mice. Data are shown as means ± SEM. *n* = 3–5 mice per group. *P < 0.05; **P < 0.01; ***P < 0.001 by unpaired Student’s *t* test.

Given that Miap410 Ab had a tremendous negative impact on microglia numbers and phenotype in SCs of EAE, we asked whether Miap410 Ab affected microglia in the SCs of nonimmunized WT (C57BL/6J) mice. Mice received Ab every other day for a total of 14 days to replicate the EAE treatment regimen. There were no differences in the number of CD45^lo^ microglia in SCs between Miap410 Ab– and MOPC-21 control Ab–treated mice (Figure 7A). CD45^lo^ microglia from Miap410 Ab–treated mice had reduced surface expression of CD11c, CD64, CCR2, and Tmem119, and significantly elevated SIRPα expression, compared with MOPC-21 mice (Figure 7B). These findings are consistent with the phenotype of microglia in EAE mice treated with Miap410 Ab (compare Figure 6I to Figure 7B). These data demonstrate that Miap410 treatment altered the SC microglia phenotype even at the baseline condition but did not induce paresis.

Because Miap410 Ab greatly affected microglia in SCs, we wondered whether microglia in *Cd47^–/–^* mice had a similar phenotype. Microglia isolated from the SCs of nonimmunized *Cd47^–/–^* mice were examined by flow cytometry. In contrast to Miap410 Ab–treated mice, the *Cd47^–/–^* mice had a significantly greater proportion of activated CD11c^hi^MHCII^hi^ microglia compared with WT mice (Figure 7, C and D). Microglia from *Cd47^–/–^* mice compared with WT also had elevated surface expression of phagocytic receptors CD36, TREM2, and Clec7A, as well as increased CCR2 (Figure 7E), and no change in expression of F4/80, CD64, CD11b, and Tmem119. The expression of SIRPα in *Cd47^–/–^* microglia was much greater than that in WT (Figure 7E), which was also observed in Miap410 treatment (Figure 7B). This difference in SIRPα expression between WT and *Cd47^–/–^* microglia is consistent with a recent study by Sato-Hashimoto and colleagues (32). Taken together, these results demonstrate that microglia in SCs were differentially affected by CD47 Ab Miap410 treatment compared with genetic deficiency in CD47.

### CD47 Ab Miap410 alters the phenotype of immune cells in spleens of nonimmunized mice.

Since blood-derived CD45^hi^ cells were scarce in the SCs of nonimmunized mice, splenocytes were examined instead. In the spleen, Miap410 Ab did not change the number of CD4^+^ or CD8^+^ T cells, γ/δ T cells, or macrophages, but surprisingly, caused a 4-fold increase in the number of Ly6G^+^ neutrophils and a small increase in monocytes (Supplemental Figure 5A). Miap410 Ab significantly reduced the total number of CD11c^+^ DCs (Supplemental Figure 5A). Further analysis showed the reduction was restricted to the CD4^+^ DCs and not the CD8^+^ DCs (Supplemental Figure 5, C–E), which replicates the data that we and others have previously reported (19, 43–46).

Miap410 Ab also significantly altered the expression of multiple surface markers on CD45^+^F4/80^+^Ly6G^–^ macrophages (see gating scheme in Supplemental Figure 6). Significant increases in SIRPα (2-fold) expression were observed on macrophages in Miap410-treated mice and to a lesser extent in CD11b, CD204 (MSR-1), Mac2, CD80, and Ly6c (Supplemental Figure 5B). In addition, Miap410 reduced the expression of the activation markers CD11c and MHCII, and the molecules involved in phagocytosis and lipid uptake, CD64 and TREM2, respectively (Supplemental Figure 5B). No differences in expression of CD36 (scavenger receptor) or Clec7A were observed. Thus, Miap410 Ab inhibition of CD47 lessened the “activation state” of splenic macrophage as compared with MOPC-21 Ab. These data demonstrate that Miap410 treatment altered splenic immune cells at baseline conditions.

As expected, Miap410 Ab treatments caused an initial drop in the erythrocyte count that returned to normal within 3 weeks (Supplemental Figure 5F), which is similar to the situation reported in human patients who had received CD47 Ab as a treatment for cancer (47). In addition, we previously reported that this same Miap410 Ab regimen altered splenic architecture with increased iron deposition (Prussian blue stain) in the red pulp area, which reflects increased uptake of RBCs by red pulp macrophages and DCs after loss of CD47 “don’t eat me” signaling molecules (19).

## Discussion

**Figure 8:**
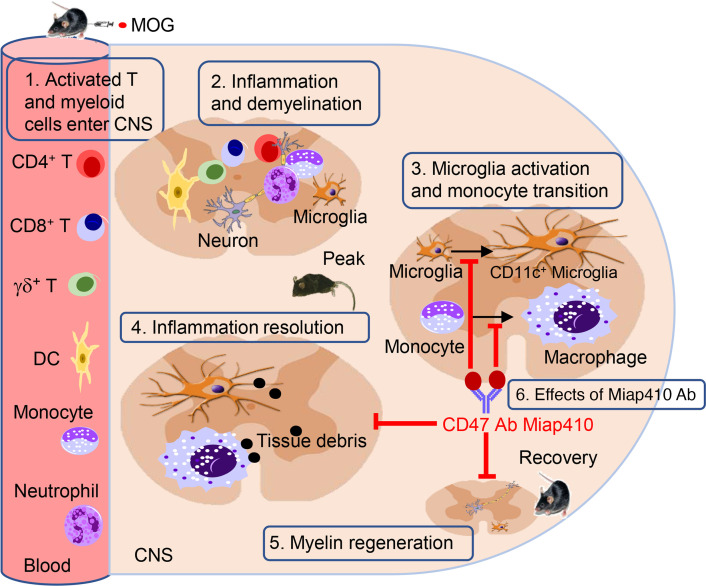
Diagram for effects of CD47 Ab Miap410 on EAE mouse model. 1. MOG immunization triggers an immune response to myelin that induces migration of T effector cells and myeloid cells into the CNS. 2. Infiltrated immune cells release proinflammatory chemokines and cytokines that promote inflammation and neuronal demyelination in the white matter of CNS, resulting in limb paresis. 3. Cytokines released by inflammatory cells activate resident microglia to express high amounts of CD11; MHCII; multiple phagocytic receptors, CD64 (FcγR1), CD36/scavenger receptor, TREM2, and Clec7A; and the autography protein LC3B-II. Infiltrated monocytes transition to macrophages and express increased amounts of F4/80 and CD64 (FcγR1). 4. Activated microglia and M2-like macrophages phagocytose the myelin fragments, damaged cells, and tissue debris, promoting resolution of the inflammation. 5. Inflammation resolves, remyelination occurs, and mice show recovery from paresis. 6. CD47 Ab Miap410 inhibits microglia activation and monocyte transition into macrophages and results in sustained SC inflammation and worsening limb paresis.

We have characterized the impact of Ab blockade of CD47 in the MOG-induced EAE model using the Miap410 Ab that we have previously reported completely inhibited the in trans CD47/SIRPα association without altering the in cis CD47/LFA-1 integrin interaction necessary for T cell recruitment (7, 14, 19, 48). The current data show that Miap410 Ab blockade of CD47 administered at the same time as MOG immunization, or at the onset of disease in the R-EAE model, reduced immune cell influx, but unexpectedly, did not attenuate disease (Figure 8).

### Impact of CD47 Ab Miap410 on immune cells in SCs in EAE.

Flow cytometric phenotyping and histological analyses of SCs at peak disease and recovery phases demonstrated that Miap410 Ab dramatically suppressed (95% reduction) the emergence of CD11c^hi^MHCII^hi^ microglia in SCs (Figure 4, B–D). This specific population of microglia has been shown to perform crucial reparative functions, including receptor-mediated phagocytosis and autophagy-mediated clearance of myelin fragments, aggregated proteins, and damaged or dead cells, and together with Mono-Macs to coordinate remyelination in multiple animal models of neuroinflammation, including EAE (31, 33, 49, 50).

A second major effect of Miap410 Ab treatment was the prevention of infiltrated monocytes’ transition to macrophages in SCs. The dramatic (94%) reduction in Mono-Macs at peak disease (Figure 3, C and J) of EAE is likely to have had a detrimental impact on resolution of inflammation and recovery given their capacity to phagocytose debris, to secrete cytokines that influence the adaptive immune cells, and to produce proresolving lipid molecules described previously by Serhan and colleagues (51–53). Further, we speculate that preventing the transition of monocytes to macrophages altered the behavior of recruited immune cells and resident glial cells and negatively affected the emergence of reparative microglia. In this way, Miap410 blockade of the transition of monocytes to Mono-Macs contributed to worsening paresis.

A third impact of Miap410 Ab was the elevated number of CD4^+^ T cells, neutrophils, monocytes, and Mono-Macs in SCs at the recovery phase (Figure 6B). The sustained inflammation observed in the “recovery” phase resembled the pathological processes that drive inflammation in relapsing EAE and Theiler’s murine encephalomyelitis virus-induced demyelinating disease models. Thus, sustained inflammation and demyelination in the CNS sets the stage for reactivation of infiltrated T cells and for naive T cells entering the inflamed SC environment to interact with and become primed by local antigen-presenting cells to initiate epitope spreading (42). Future studies are needed to determine whether neoantigens or MOG 35–55 peptide, or both, induced reactivation of resident CD4^+^ T cells and perpetuated the inflammatory response observed in Miap410 Ab–treated mice.

### Microglia CD11c^hi^MHCII^hi^ phenotype aids in recovery in EAE.

Microglia undergo a rapid transformation in response to CNS pathology that promotes both injury and repair (4, 31). This transformation occurs as a continuum that entails intermediate stages propelled by changes in gene expression, proliferation, migration, metabolism, secretome, phagocytosis, and death. A recent study reported that remyelination was mediated by proinflammatory microglia undergoing necroptosis death followed by repopulation of white matter microglia with a reparative phenotype (31). CD11c^+^ microglia have been shown to play a critical role to repair or remove damaged myelin in the white matter of the CNS (32, 38–40). Our results indicate that Miap410 Ab inhibited the emergence of microglia equipped with activation markers CD11c, MHCII, and CCR2 and multiple surface receptors for removal of myelin debris, including CD64 (FcγR1); CD36, which is a scavenger receptor; TREM2, which binds lipids and promotes microglial expansion upon CNS insults (54, 55); and Clec7A (Dectin-1), which is a pattern recognition receptor (Figures 4–6). Miap410 Ab also inhibited LC3B-II–mediated autophagy (Figure 5C).

The mechanism by which CD47 Ab blocked the emergence of prophagocytic Tmem119^+^CD45^lo^ microglial cells is not known. Future studies are needed to address this question. Thus, we propose a model in which the mechanism of Miap410 Ab is either indirect or direct, or both. The indirect mechanism would be through blocking the transition of blood-derived monocytes into Mono-Macs or into DCs that physically interact with microglia, or through secretion of soluble factor(s) that ignite a program driving the emergence of CD11c^hi^MHCII^hi^ microglia with reparative functions, which were prominent during recovery in control MOPC-21 Ab–treated mice (Figures 5 and 6). The data in support of this model are that treatment of mice with Miap410 mAb blocked 94% of blood monocyte transition to Mono-Macs and significantly blocked the influx of CD4^+^, CD8^+^, and γ/δ T cells at peak disease (Figure 3C). Alternatively, Miap410 Ab could have had a direct effect by gaining access into the CNS through encephalomyelitis-induced loss in blood-brain barrier that occurs in MOG-induced EAE (56). Miap410 Ab in this setting also caused significantly increased surface expression of SIRPα and significantly reduced the surface expression of several other molecules involved in phagocytosis, lipid uptake, and cell adhesion and trafficking in CD11c^hi^MHCII^hi^ microglia (Figure 5B and Figure 6I) that impaired animal recovery in models of neuroinflammation. The mechanism(s) of Miap410 Ab altering the phenotype of immune cells are likely complex, involving both direct and indirect effects, and at the level of transcriptional gene control of the other molecules we have monitored in this project. Importantly, Miap410 Ab also reduced the expression of Ly6c, CD11c, CD64, and CCR2 in Mono-Mac at peak disease (Figure 3L). Sato-Hashimoto and colleagues recently concluded that microglia SIRPα suppresses the induction of CD11c^+^ microglia that accelerate repair of damaged white matter in a model of cuprizone-induced demyelination (32). Similarly, mice with genetic deletion of SIRPα show superior phagocytic clearance of myelin debris in a mouse model of Wallerian degeneration (57). In our study, we observed that Miap410 Ab treatment increased SIRPα protein expression on microglia, significantly reduced the number of CD11c^hi^MHCII^hi^ microglia, and decreased their surface expression of CD11c, MHCII, CD64, CCR2, CD36, TREM2, and Clec7A (Figures 4–7). Interestingly, *Cd47^–/–^* microglia also expressed a dramatically greater amount of SIRPα as compared with WT mice (Figure 7); however, a significant proportion of the *Cd47^–/–^* microglia were CD11c^hi^MHCII^hi^ and showed elevated expression of CCR2, CD36, TREM2, and Clec7A. Taken together, these data suggest that genetic deletion of either SIRPα or CD47 induces the emergence of CD11c^hi^MHCII^hi^ microglia in SCs, whereas treatment with a CD47 Ab had the opposite effect and prevented their emergence.

### Targeting CD47 in EAE.

The 70% inhibition of T cell recruitment by Miap410 Ab at peak disease in MOG-induced EAE was in line with our prior in vitro studies that Ab blockade of CD47 expressed in human or murine endothelial cells and in T cells, or mice genetically deficient in CD47 in an atherosclerosis model, exhibit significantly reduced T cell transendothelial migration (7, 17, 48). Regarding other CD47-targeted treatments in EAE, Gao and colleagues (20) reported that a CD47-Fc chimera had impressive beneficial effects in EAE; however, the authors reported only a reduction in Th17 cell influx and no other description of immune cell phenotypes in CD47-Fc chimera–treated mice. The mode of action for the CD47-Fc is different than use of an intact Ab to block CD47. The CD47-Fc chimera specifically binds to SIRPα, not to CD47. In addition, SIRPα expression is restricted to myeloid cells and neurons, and as just discussed, deletion of SIRPα, or blocking its interaction with CD47, induces the emergence of reparative CD11c^+^ microglia (32). Unfortunately, the authors (20) did not provide structural information for the CD47-Fc chimera and whether the Fc portion of the chimera could engage and activate FcγRs. We also point out that *Cd47*^–/–^ mice, and mice expressing a mutant, truncated, nonsignaling SIRPα, were completely protected in EAE because these mice were unable to generate a sufficient effector T cell response to MOG peptide, and hence histopathology revealed no inflammation or myelin loss had occurred in the SCs (17, 58). In summary, blockade of CD47 by Miap410 Ab inhibited the emergence of reparative CD11c^hi^MHCII^hi^Tmem119^+^ microglia, prevented monocyte transition to reparative Mono-Macs, and as a result, prevented resolution of inflammation and recovery that worsened paresis in EAE.

We also note that Miap410 treatment of nonimmunized mice caused a transient anemia at day 7 in mice that recovered within 3 weeks (Supplemental Figure 5F), consistent with our previous report (19). Anemia is a common reported side effect for humanized Ab Hu5F9 against CD47 used to treat patients with various solid and blood tumors (59). It is unclear what the impact of this transient anemia would have had in the EAE model because the amount of inflammation was less than that observed in the control MOPC-21 Ab mice at peak disease (DPI 15), and the worsening of disease in the recovery phase was likely due to sustained antigen-induced) or neoantigen-induced immune responses. Given the rather blunt outcomes, it is of course hard to know whether more subtle changes may have occurred in distinct immune populations under similar conditions, but in general there is quite a hemoglobin reserve in most mammals (60–62). Nonetheless, we do not discount the potential contribution of the anemia to the worsening paresis by Miap410.

We also considered whether the impaired recovery in EAE was related to an FcγR binding effect of Miap410 Ab. Miap410 Ab is a murine IgG1 isotype that can bind to FcγRs on myeloid cells, resulting in their activation. Our data do not address whether an FcγR binding effect of this Ab occurred or contributed to inflammation in Miap410-treated mice; however, a role for FcγR-dependent myeloid cell activation did not seem pathogenic at peak of disease because Miap410-treated mice had significantly less inflammation in the SCs as compared with control Ab (Figure 3). During the recovery phase in Miap410 mice, the most abundant cell type was the CD4^+^ T cell, which suggests a MOG-driven or a neoantigen-driven immune reaction (Figure 6). However, an uptick in neutrophils and monocytes in some, but not all, mice was observed; thus, we do not discount an FcγR-mediated myeloid cell contribution.

These findings in EAE models bring attention to the potential for off-target effects of blocking CD47 in cancer immunological therapeutics. Abs against CD47 such as Hu5F9-G4 (magrolimab) that block the CD47/SIRPα axis are in multiple clinical trials as a cancer immunotherapeutic in combination with other cancer therapeutics, including rituximab; have shown efficacy in certain human hematological cancers (21–25); and more recently have been shown to reduce inflammation in carotid arteries in a very small retrospective analysis of patient trial data (26). Our results identify potential detrimental side effects on the CNS by treatments using CD47-blocking Ab that should be considered when treating patients with demyelinating disease.

## Methods

### Mice.

Female C57BL/6J (Stock000664|B6) and SJL (Stock 000686|SJL) 8-week-old mice were purchased from The Jackson Laboratory. Mice with total deficiency in *Cd47* (Stock 003173|IAP-C57BL/6J) (63) were purchased from The Jackson Laboratory and were used to establish a colony for use in this study. Upon arrival at our animal facility, the mice were acclimatized for 7 days prior to enrollment in EAE studies. Mice were housed in a climate-controlled room with a 12-hour light/12-hour light dark cycle and food and water ad libitum.

### Materials.

PBS and Dulbecco’s phosphate-buffered saline (DPBS) with or without Ca^2+^ and Mg^2+^ (DPBS^+^, DPBS^–^) were purchased from Lonza. Incomplete Freund’s adjuvant (IFA) was purchased from BD Biosciences. *Mycobacterium tuberculosis* (*M. tuberculosis*, H37Ra) was from Difco Laboratories. MOG peptide aa 35 to 55 (M-E-V-G-W-Y-R-S-P-F-S-RO-V-H-L-Y-R-N-G-K), corresponding to the mouse protein sequence, was purchased from Genemed Synthesis. Proteolipid protein peptide aa 139–151 (H-S-L-G-K-W-L-G-H-P-D-K-F) (PLP), corresponding to the mouse protein sequence, was purchased from Genemed Synthesis. Pertussis toxin (PT) was purchased from List Biological Laboratories. Digitonin, PMA, and ionomycin were purchased from MilliporeSigma. The following murine mAbs were purified, azide-free IgG produced for in vivo use, and purchased from Bio X Cell: Miap410, IgG1κ (clone BP0283); and MOPC-21, IgG1κ (clone BP0083). All Abs used for flow cytometry are listed in Table 1.

### Immune cell isolation from SCs by Percoll gradient centrifugation.

Mice were sacrificed and perfused through the left ventricle with 10 mL of ice-cold PBS without Ca2^+^ and Mg2^+^ (PBS^–^). The SC was flushed out by hydraulic extrusion with a 5 mL syringe containing a pipet tip (64). SC handling time was significantly reduced to a few minutes compared with SC dissection, likely decreasing cell damage and loss, thus improving immunohistochemical analysis. SCs were passed through 70 μm cell strainers (Falcon), and the cell suspension mixed with stock isotonic Percoll (SIP) (9 parts of Percoll and 1 part of 10X HBSS without Ca^2+^ and Mg^2+^ [HBSS^–^]) to create 30% SIP. The 30% SIP was layered on a 70% SIP solution and centrifuged at 500*g* for 30 minutes at room temperature. Immune cells in 2 mL of the 30%–70% SIP interphase were collected, diluted to 10 mL with HBSS^–^ to wash out Percoll, and centrifuged as before, and the pellet was resuspended into FACS buffer. FACS wash and stain buffers consisted of PBS^–^ with 0.5% BSA and 0.02% sodium azide on ice.

### RBC counts by Coulter analyzer.

Mouse blood was collected into 100 μL microvettes containing K_3_EDTA anticoagulant (Sarstedt Ag). Blood was diluted 1:50,000 in ISOTON II diluent (Beckman Coulter), and RBC counts were determined in triplicate by a Coulter Z2 series particle and size analyzer (Beckman Coulter) equipped with a 100 μm aperture tube and set up for RBC counts. The normal C57BL/6J RBC count is 9.66 × 10^9^/mL (65).

### Multicolor immunofluorescence phenotyping of immune cells and FACS analysis.

Single-cell suspensions of immune cells were analyzed by flow cytometry using optimized concentrations of Ab directly labeled by fluorescent dyes purchased from BioLegend and BD Biosciences (Table 1). Propidium iodine viability dye (BioLegend) was included to exclude all nonviable cells from the analysis. Anti-CD16 and anti-CD32 mAbs were added to the Ab master mix in FACS buffer to block nonspecific Ab binding to cell FcγRs. Flow cytometry was performed using an 8-color FACSCanto instrument (BD Biosciences). All flow cytometry data were analyzed using FlowJo software version 10.6 (BD Biosciences). The gating strategy used for analysis of SC and spleen immune cells is depicted in Supplemental Figure 1.

### Detection of intracellular IFN-γ and IL-17A.

Intracellular IFN-γ and IL-17A were detected in PMA, ionomycin, and brefeldin A stimulated immune cells isolated from SCs of mice by flow cytometry as we have previously reported (19).

### LC3B-II detection in cells and digitonin permeabilized protocol.

Membrane-bound lipidated LC3B-II was measured in cells after mild digitonin permeabilization to extract cytosolic LC3 using modification of a previously published method (33, 66). Cells were incubated at room temperature for 5 minutes in PBS^+^ containing digitonin (50 μg/mL), washed, fixed in 4% PFA in PBS^–^ for 10 minutes at room temperature, washed twice in PBS^–^, and stained with mAb against LC3B, with fluorescence quantified by flow cytometry.

### Induction of EAE.

EAE was induced as described (17). In brief, acclimatized, 9-week-old, female C57BL/6J mice were immunized subcutaneously in the flank with 100 μg MOG emulsified 1:1 in IFA supplemented with 4 μg/mL of *M. tuberculosis* (H37Ra; Difco Laboratories). Animals received 200 ng by i.p. injection of PT on days 0 and 2 postimmunization. R-EAE was induced in acclimatized 9-week-old female SJL/J mice by immunization subcutaneously with 100 μL (50 μg) of PLP emulsified 1:1 in IFA containing 4 μg/mL of *M. tuberculosis*. PLP-immunized mice did not receive PT. Immunized mice were observed daily starting day 8 DPI. EAE and R-EAE clinical signs were scored as follows: grade 0, no disease; grade 1, limp tail or isolated weakness; grade 2, partial hind limb paralysis; grade 3, total hind limb paralysis; grade 4, total hind limb and partial forelimb paralysis; grade 5, moribund or dead animal.

### Spinal cord histology.

The lumbar segments of SCs were removed for histological studies, and the remaining portion of SC was processed for flow cytometric analysis. SC specimens were fixed for 24 hours at 4°C in 4% PFA-PBS and then embedded in paraffin. Five-micron transverse sections of SC specimens were cut with a microtome (Leica Microsystems) and stained with Luxol blue using a commercial kit following the manufacturer’s directions (IHC World) and counterstained with a cresyl violet solution, or with an Ab against CD4, macrophages (Mac2), or microglia (Tmem119) (Table 2). Images of SC sections were obtained with an upright phase contrast microscope (Nikon Microphot-FX4, Nikon Instruments) equipped with a high-resolution color camera and 4x (plan4/0.13 NA) and 20x objectives (planApo20/0.75 NA). Blinded selection of the images was performed.

### Statistics.

Statistical analyses used Prism 8 (GraphPad Software). Data are expressed as mean ± SEM and *n* samples analyzed. Statistical analyses were by paired or unpaired 2-tailed Student’s *t* test between 2 groups and by 1-way ANOVA with Tukey’s post hoc test for 3 or more groups, with *P* < 0.05 considered significant.

### Study approval.

All animal research was approved by and performed in accordance with the guidelines of the Institutional Animal Care and Use Committee at the Mass General Brigham (formerly Brigham and Women’s Hospital) and with the US NIH animal research guidelines, as set forth in the *Guide for the Care and Use of Laboratory Animals* (National Academies Press, 2011).

## Author contributions

HW, GN, LW, LLL, SN, ASM, and FWL designed the research, and HW and GN performed the experiments, selected the histological images in a blinded fashion, and analyzed the data. FWL and HW wrote the manuscript.

## Supplementary Material

Supplemental data

## Figures and Tables

**Table 1:**
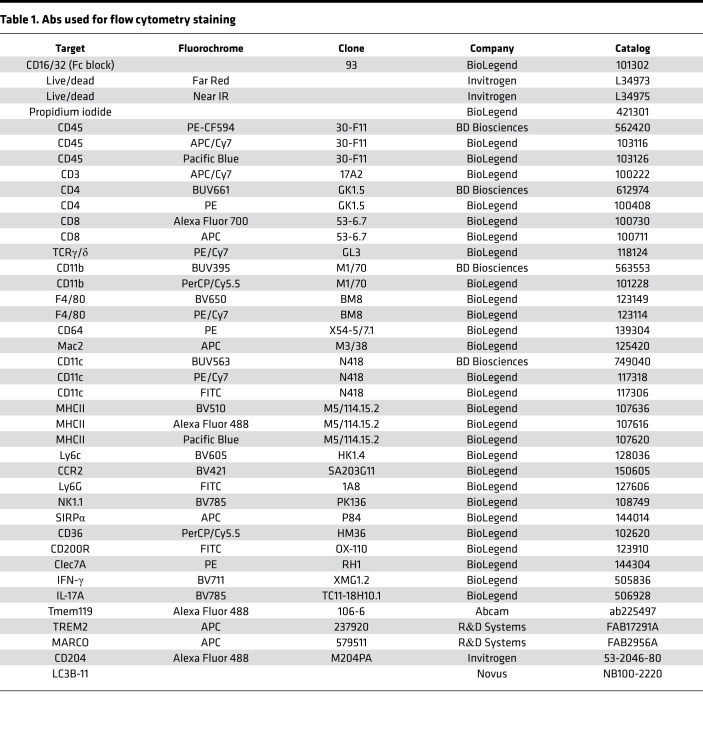
Abs used for flow cytometry staining

**Table 2:**
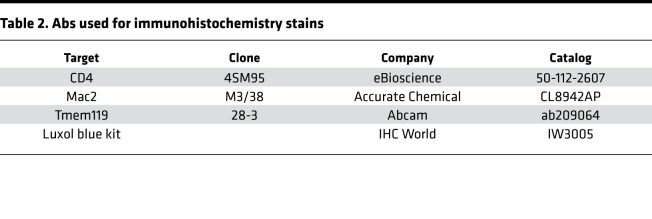
Abs used for immunohistochemistry stains

## Version 1 09/30/2021

In-Press Preview

## Version 2 11/08/2021

Electronic publication
