# Supplementary material for: CD47 antibody blockade suppresses microglia-dependent phagocytosis and monocyte transition to macrophages, impairing recovery in EAE
Source: JCI Insight. 2021 Nov 8;6(21):e148719. doi: 10.1172/jci.insight.148719 (PMC8663579; doi:10.1172/jci.insight.148719)
Supplement: Supplemental data [file jciinsight-6-148719-s033.pdf]

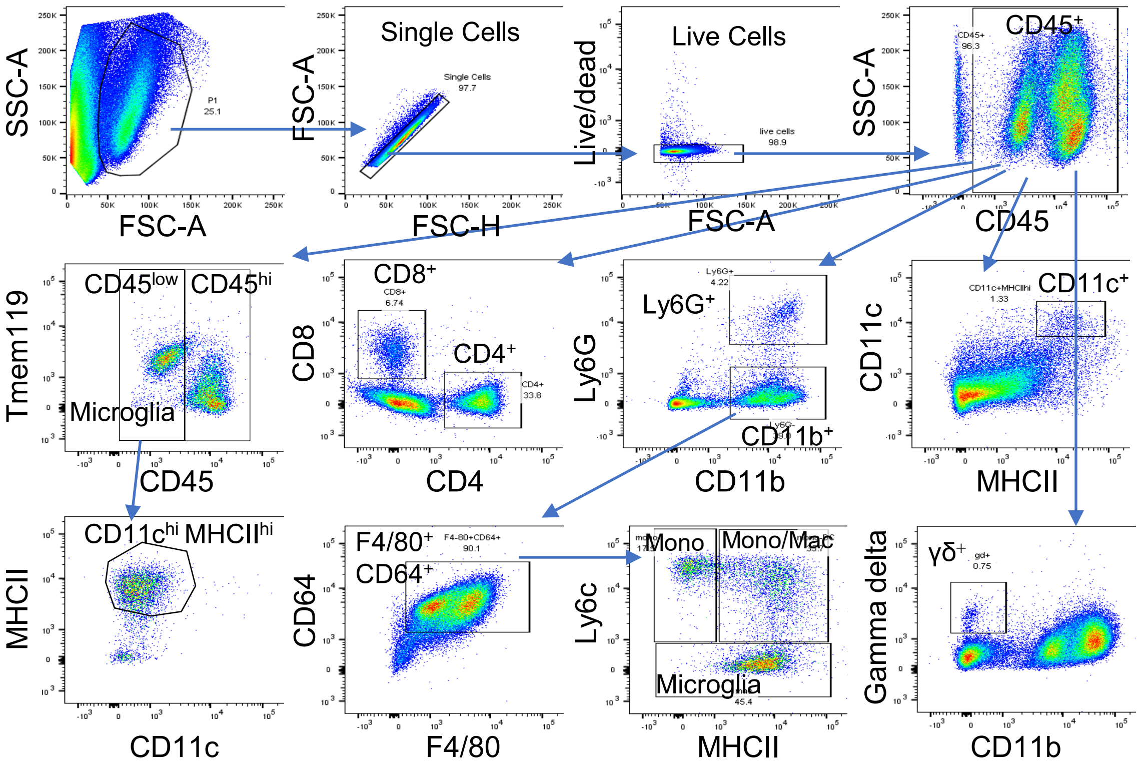

Supplemental Figure 1. The gating scheme for FACS analysis immune cells isolated at peak disease from the spinal cord of MOG peptide immunized mice.

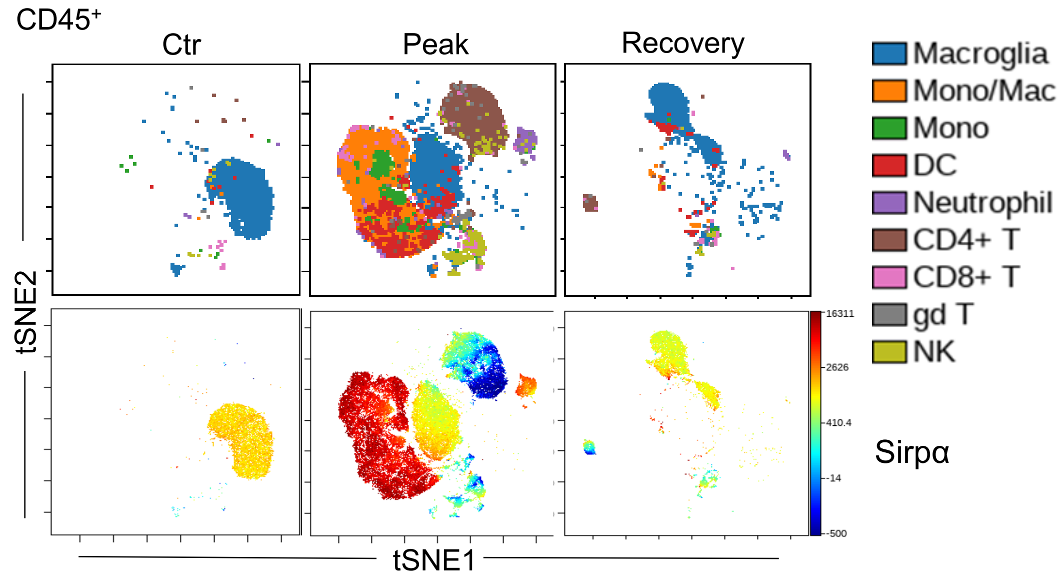

Supplemental Figure 2. Sirpα expression on immune cells in MOG-induced EAE mice at peak. Sirpα expression on immune cells in the spinal cord by t-SNE.

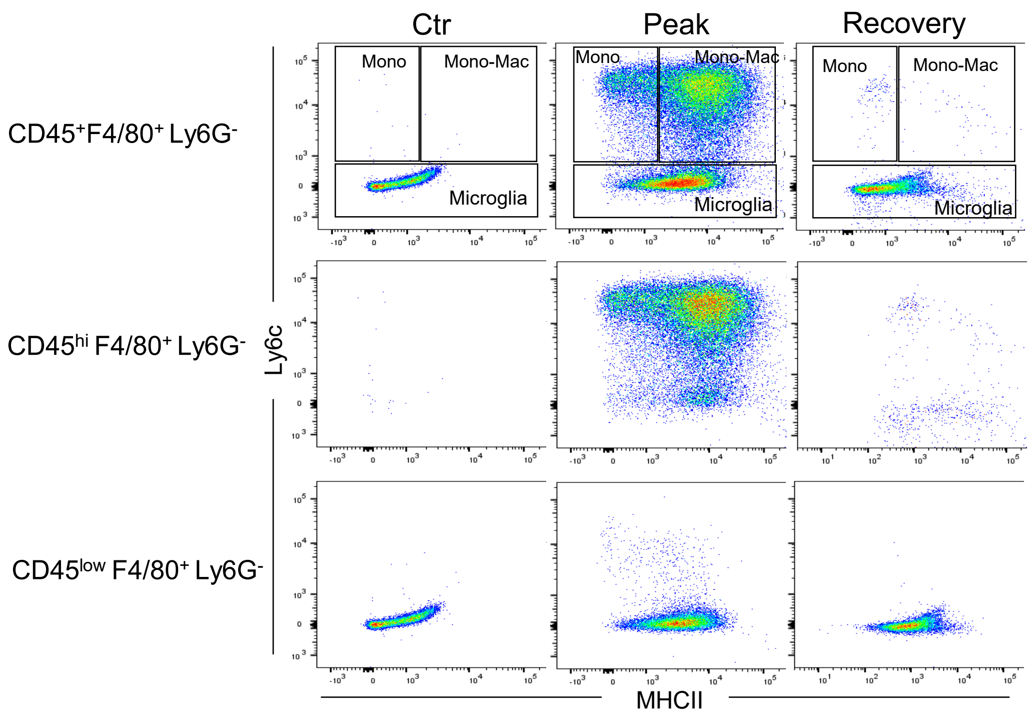

Supplemental Figure 3. CD45^{high} (blood derived) monocytes, monocyte-derived macrophages (Mono-Mac), and CD45^{low} microglia cells in the spinal cord of MOG peptide-induced EAE mice at day 16 (peak) and day 30 (recovery). Only CD45^{low} microglia were detected in non-immunized control (Ctr) mice.

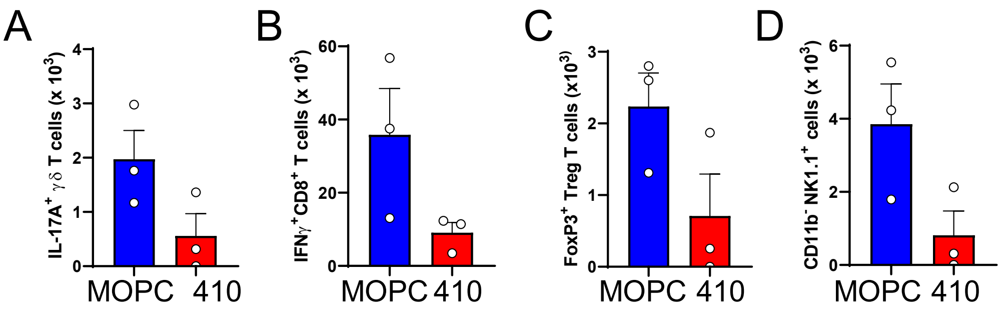

Supplemental Figure 4: (A – D) T cell subsets present at low numbers in Miap410 Ab treated compared to MOPC-21 control treated mice. N=3 mice per group. No statistical significances between Ab treatments was found by the unpaired Student's t test.

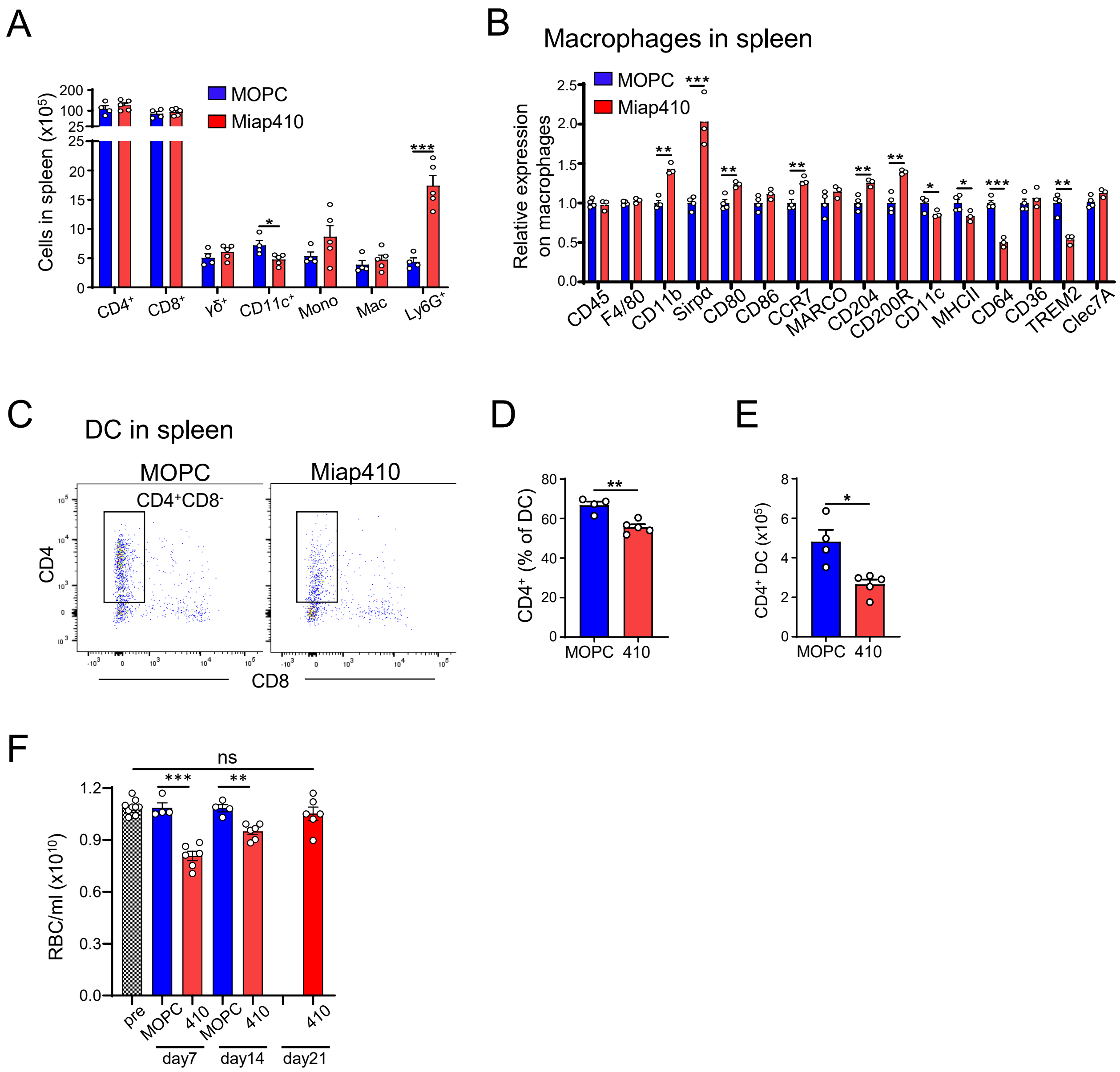

Supplemental Figure 5. CD47 Ab Miap410 treatment of non-immunized mice significantly alters the phenotype of splenic immune cells. (A) Total number of different immune cell subtypes in spleen. (B) Relative protein expression on macrophages in spleen. (C) Representative plots of $CD4^+$ dendritic cells (DC) in spleen. (D, E) Percentage of DCs that are $CD4^+$ and total number of $CD4^+$ DCs in spleen. (F) RBC counts in CD47 Ab treated mice. Data are shown as means ± SEM. n= 3 - 6 mice per group. * $p < 0.05$; ** $p < 0.01$; *** $p < 0.001$ by unpaired Student's t test.

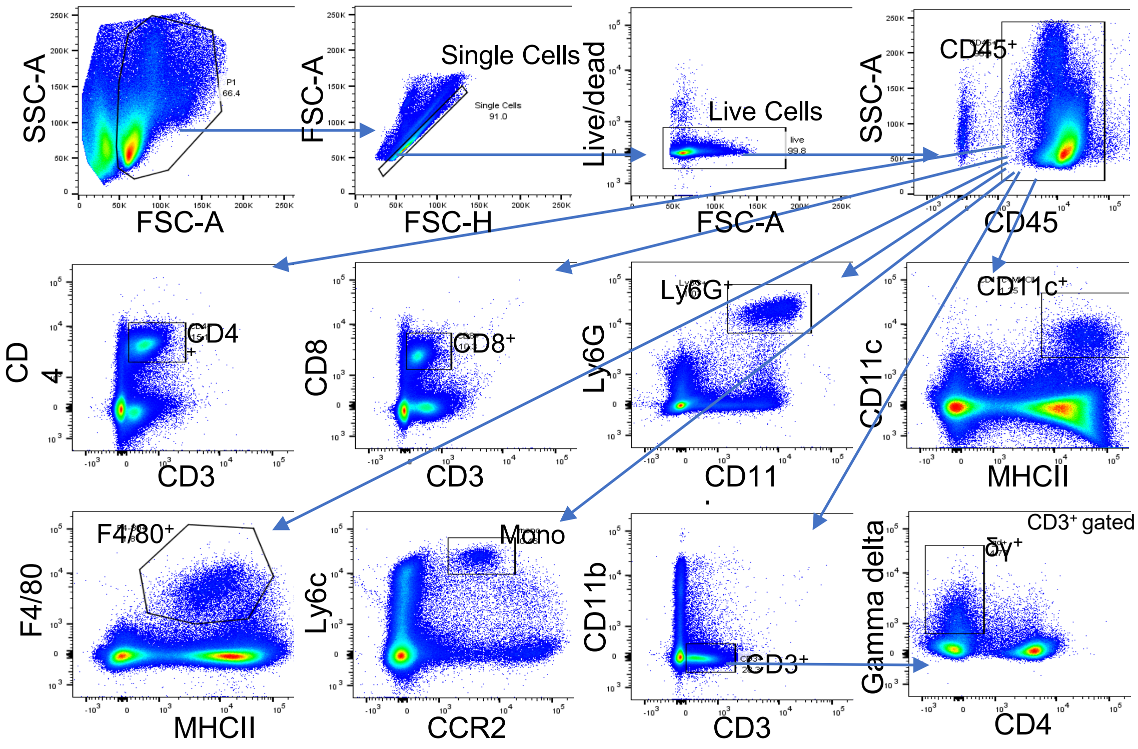

Supplemental Figure 6. Gating scheme for FACS analysis of spleen from mice.
